# Supplementary material for: Transcription stress at telomeres leads to cytosolic DNA release and paracrine senescence
Source: Nat Commun. 2024 May 14;15:4061. doi: 10.1038/s41467-024-48443-6 (PMC11094137; doi:10.1038/s41467-024-48443-6)
Supplement: Supplementary file 1 — Supplementary Information [file 41467_2024_48443_MOESM1_ESM.pdf]

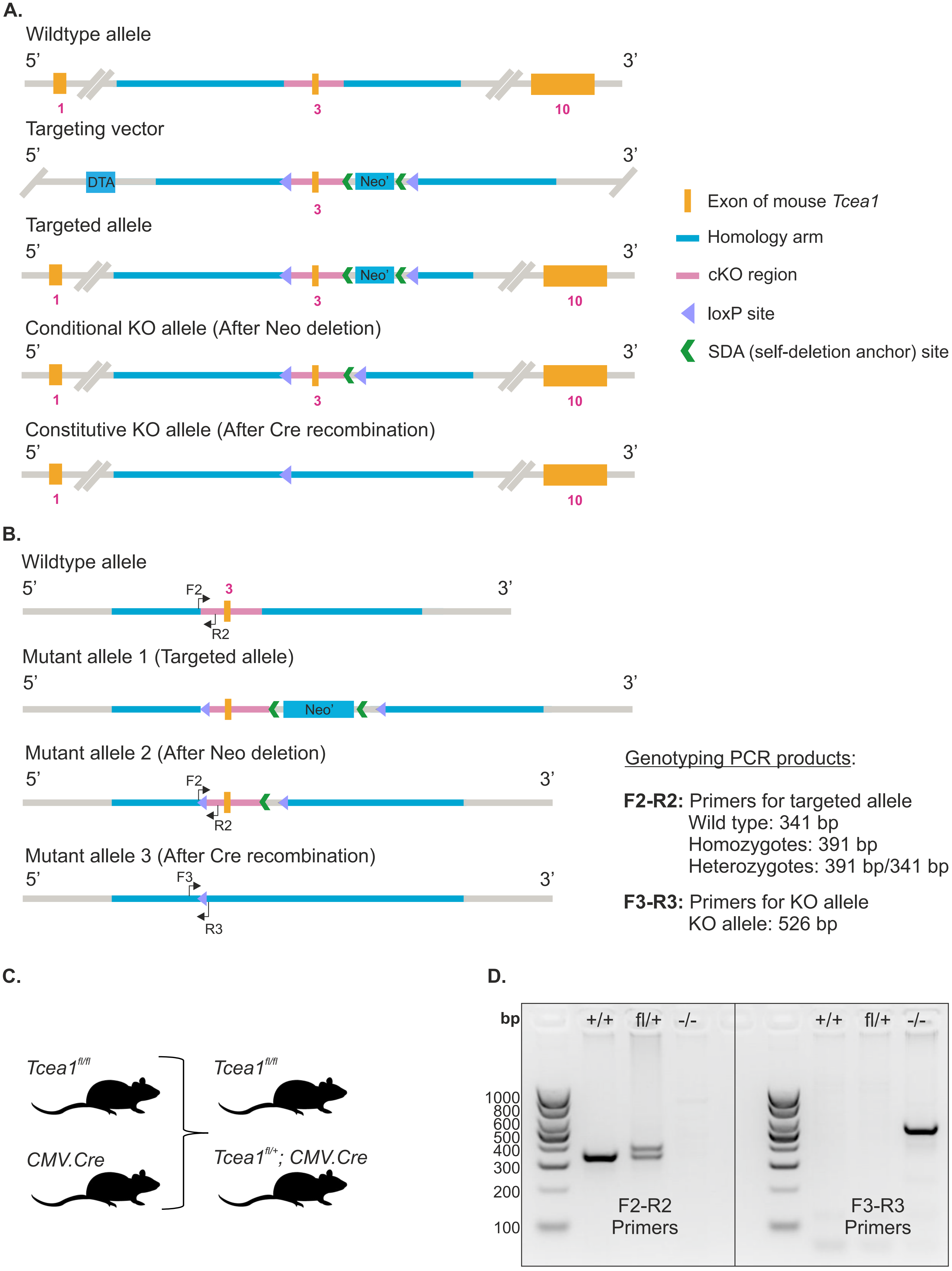
A.
Wildtype allele
5'
3'
1
3
10
Targeting vector
DTA
Neo'
Targeted allele
Conditional KO allele (After Neo deletion)
Constitutive KO allele (After Cre recombination)
Exon of mouse Tcea1
Homology arm
cKO region
loxP site
SDA (self-deletion anchor) site
B.
Wildtype allele
F2
R2
Mutant allele 1 (Targeted allele)
Mutant allele 2 (After Neo deletion)
Mutant allele 3 (After Cre recombination)
F3
R3
Genotyping PCR products:
F2-R2: Primers for targeted allele
Wild type: 341 bp
Homozygotes: 391 bp
Heterozygotes: 391 bp/341 bp
F3-R3: Primers for KO allele
KO allele: 526 bp
C.
Tcea1fl/fl
CMV.Cre
Tcea1fl/fl
Tcea1fl/+; CMV.Cre
D.
bp
+/+
fl/+
-/-
1000
800
600
500
400
300
200
100
F2-R2
Primers
F3-R3
Primers

Supplementary Figure 1. Generation of *Tcea1*$^{-/-}$ MEFs. (A). Targeting strategy of exon 3 of the *Tcea1* gene, on mouse chromosome 1. **(B)**. PCR genotyping design of successful Cre recombination for knock-out allele generation. **(C)**. *Tcea1*$^{fl/fl}$;CMV-Cre mouse breeding scheme. **(D)**. Agarose gel confirmation of *Tcea1* allele excision by PCR. Data analysis was performed using two-tailed Student's *t*-test. All data are presented as mean values ± SEM. Unless otherwise indicated, n = biologically independent experiments and scale bars are set at 5μm.

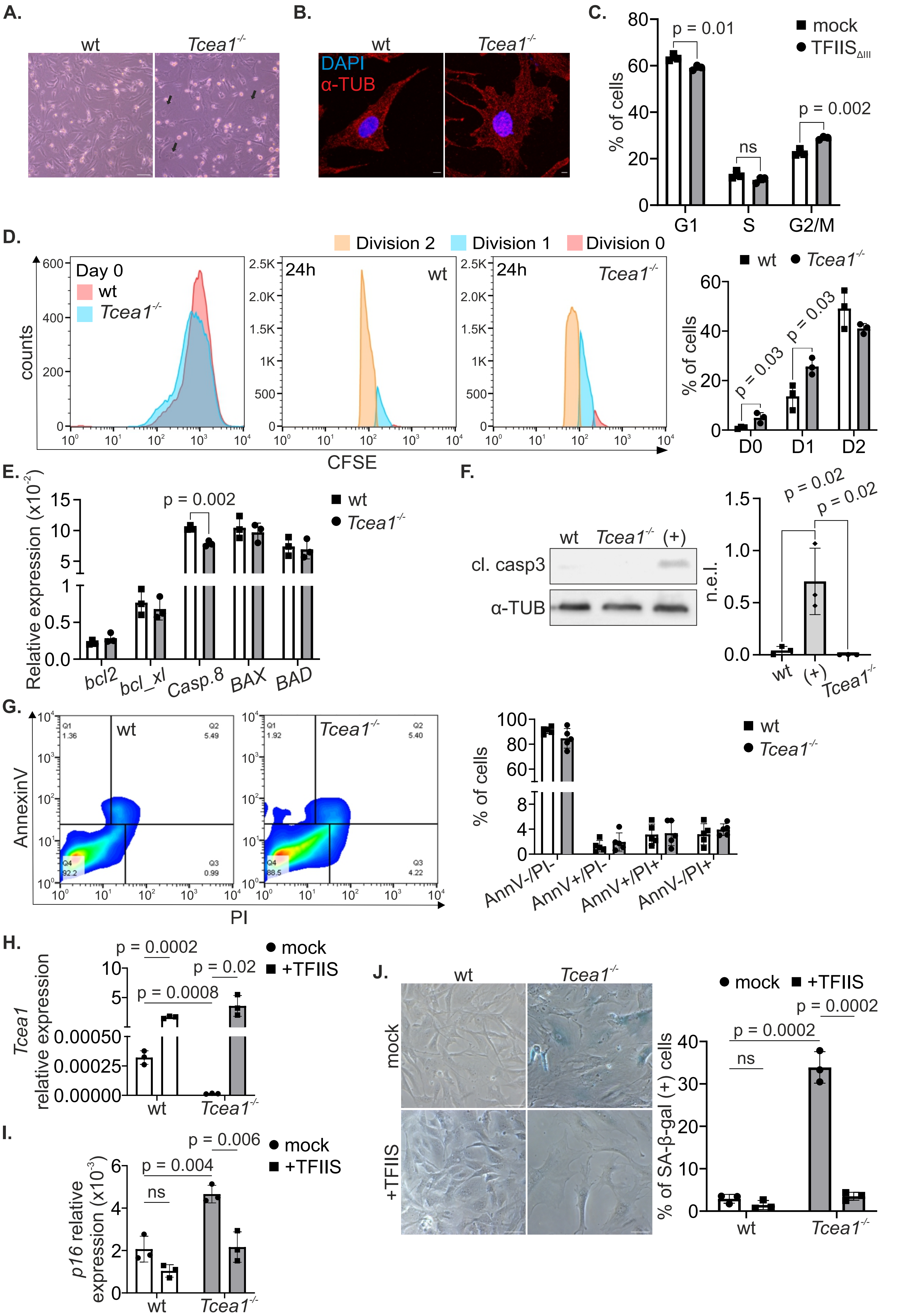
A.
wt
Tcea1-/-
B.
wt
Tcea1-/-
DAPI
α-TUB
C.
p = 0.01
ns
p = 0.002
mock
TFIISΔIII
% of cells
G1
S
G2/M
D.
Division 2
Division 1
Division 0
Day 0
wt
Tcea1-/-
counts
24h
CFSE
% of cells
p = 0.03
p = 0.03
D0
D1
D2
E.
Relative expression (x10-2)
p = 0.002
wt
Tcea1-/-
bcl2
bcl_xl
Casp.8
BAX
BAD
F.
cl. casp3
α-TUB
(+)
n.e.l.
p = 0.02
p = 0.02
G.
AnnexinV
PI
Q1
Q2
Q3
Q4
1.36
5.49
0.99
92.2
1.92
5.40
4.22
88.5
% of cells
AnnV-/PI-
AnnV+/PI-
AnnV+/PI+
AnnV-/PI+
H.
Tcea1 relative expression
p = 0.0002
p = 0.02
p = 0.0008
mock
+TFIIS
I.
p16 relative expression (x10-3)
p = 0.006
p = 0.004
ns
J.
mock
+TFIIS
% of SA-β-gal (+) cells
p = 0.0002
p = 0.0002
ns

Supplementary Figure 2. Senescent characteristics of *Tcea1*$^{-/-}$ MEFs. (A). Representative images of wt and *Tcea1*$^{-/-}$ MEFs. Arrows indicate the senescent-like characteristics of *Tcea1*$^{-/-}$ cells. Scale bar is set at 20μm. **(B)**. Immunostaining of α-TUBULIN (α-TUB) in wt and *Tcea1*$^{-/-}$ MEFs. **(C).** Cell cycle profiling using FACS analysis of untransfected (mock), TFIIS-overexpressing (+TFIIS) or TFIIS$^{\Delta III}$-overexpressing (+TFIIS$^{\Delta III}$) wt MEFs. The graph shows the percentage of cells in each cell cycle phase (n = 3). **(D).** Carboxyfluorescein Diacetate Succinimidyl Ester (CFSE) proliferation assay and representative images of FACS analysis of wt and *Tcea1*$^{-/-}$ MEFs at day 0 and after 24h. The graph represents the percentage of cells that divided 0 to 2 times (D0-D2), during a 24h time-period, according to CFSE fluorescence intensity analysis (n = 3). **(E)**. *Bcl2*, *Bcl_xl*, *Casp.8*, *BAX*, and *BAD* mRNA levels in wt and *Tcea1*$^{-/-}$ MEFs (n = 3). **(F)**. Cleaved caspase 3 (cl. casp3) in wt and *Tcea1*$^{-/-}$ MEFs whole-cell extracts. Protein extracts from camptothecin-treated (10μM for 16h) wt MEFs were used as positive control (+) (n = 3). α-TUB was used to normalize protein expression levels (n.e.l.). **(G)**. Fluorescence cytometry representative plots for AnnexinV/Propidium Iodide (PI) in wt and *Tcea1*$^{-/-}$ MEFs. The graph depicts the percentage of PI+, AnnexinV+, PI+/AnnexinV+ and PI-/AnnexinV- wt and *Tcea1*$^{-/-}$ MEFs (n=5). *Tcea1* **(H)** and *p16* **(I)** mRNA levels in untreated (mock) and TFIIS-overexpressing wt and *Tcea1*$^{-/-}$ MEFs (n=3). **(J)**. SA-β-gal assay in untreated (mock) and TFIIS-overexpressing wt and *Tcea1*$^{-/-}$ MEFs (n = 3). Scale bar is set at 20μm. Data analysis was performed using two-tailed Student's *t*-test. All data are presented as mean values ± SEM. Unless otherwise indicated, n = biologically independent experiments and scale bars are set at 5μm. Source data are provided as a Source Data file.

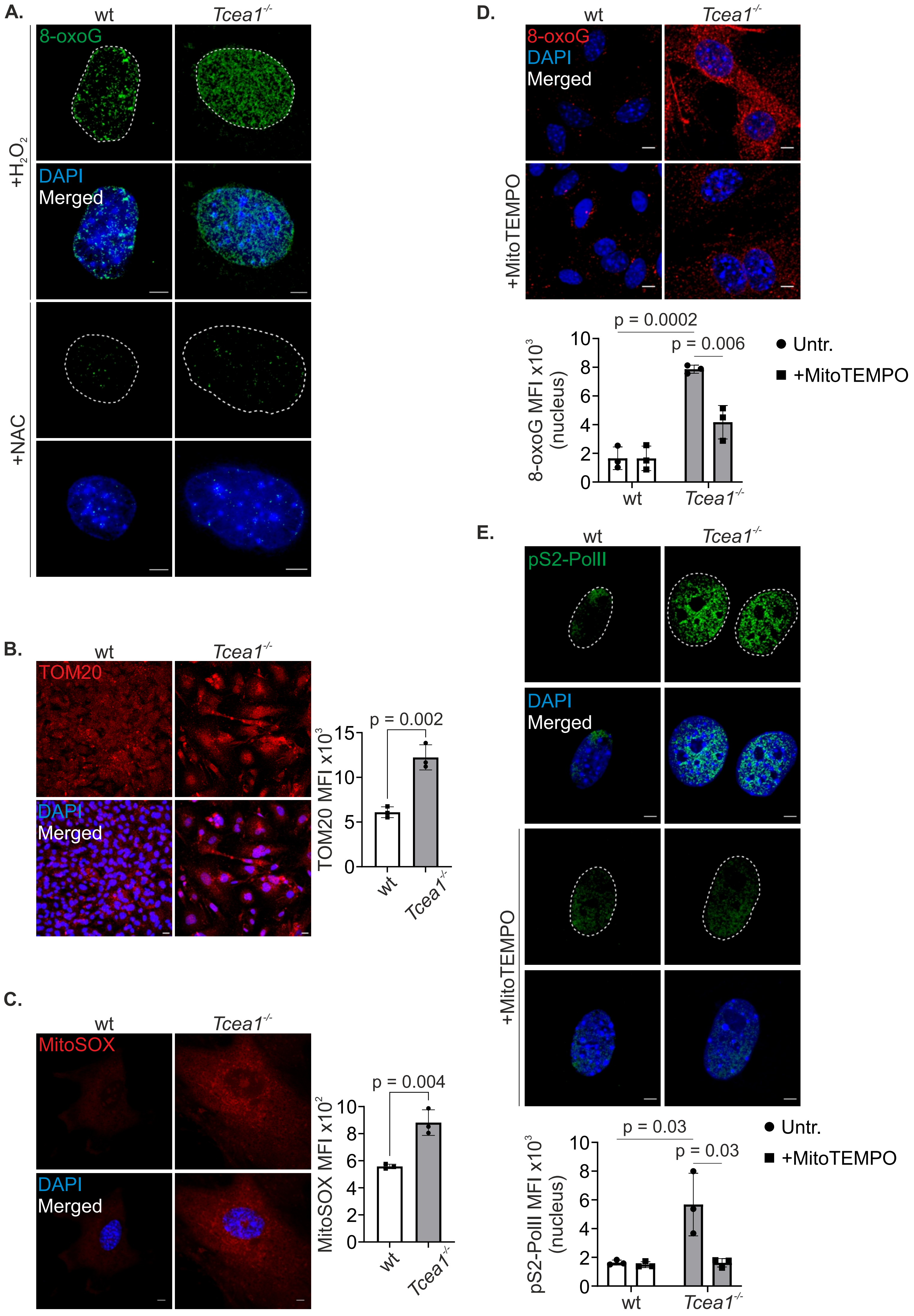

A.
wt
Tcea1-/-
8-oxoG
DAPI
Merged
+H2O2
+NAC
B.
wt
Tcea1-/-
TOM20
DAPI
Merged
p = 0.002
TOM20 MFI x10^3
0
5
10
15
wt
Tcea1-/-
C.
wt
Tcea1-/-
MitoSOX
DAPI
Merged
p = 0.004
MitoSOX MFI x10^2
0
2
4
6
8
10
wt
Tcea1-/-
D.
wt
Tcea1-/-
8-oxoG
DAPI
Merged
+MitoTEMPO
p = 0.0002
p = 0.006
8-oxoG MFI x10^3 (nucleus)
0
2
4
6
8
10
wt
Tcea1-/-
Untr.
+MitoTEMPO
E.
wt
Tcea1-/-
pS2-PolII
DAPI
Merged
+MitoTEMPO
p = 0.03
p = 0.03
pS2-PolII MFI x10^3 (nucleus)
0
2
4
6
8
10
wt
Tcea1-/-
Untr.
+MitoTEMPO

Supplementary Figure 3. Dysfunctional mitochondria in *Tcea1*$^{-/-}$ MEFs. (A). Immunostaining of 8-Oxoguanine (8-oxoG) in H_2O_2-treated and NAC-treated wt and *Tcea1*$^{-/-}$ MEFs (n = 3). **(B)**. Immunofluorescence of TOM20 in wt and *Tcea1*$^{-/-}$ MEFs. The graph depicts the TOM20 MFI in the cell cytoplasm (n = 3). **(C)**. Immunofluorescence of MitoSOX in wt and *Tcea1*$^{-/-}$ MEFs. The graph depicts the MitoSOX MFI in the cell cytoplasm (n = 3). Immunofluorescence of 8-oxoG **(D)** and Serine 2 (pS2)-phosphorylated RNAPII (pS2-PolII) **(E)** in wt and *Tcea1*$^{-/-}$ MEFs, in the absence or presence of MitoTEMPO. Each graph depicts the 8-oxoG and pS2-PolII MFI per cell nucleus (n = 3). Data analysis was performed using two-tailed Student's *t*-test. All data are presented as mean values ± SEM. Unless otherwise indicated, n = biologically independent experiments and scale bars are set at 5μm. Source data are provided as a Source Data file.

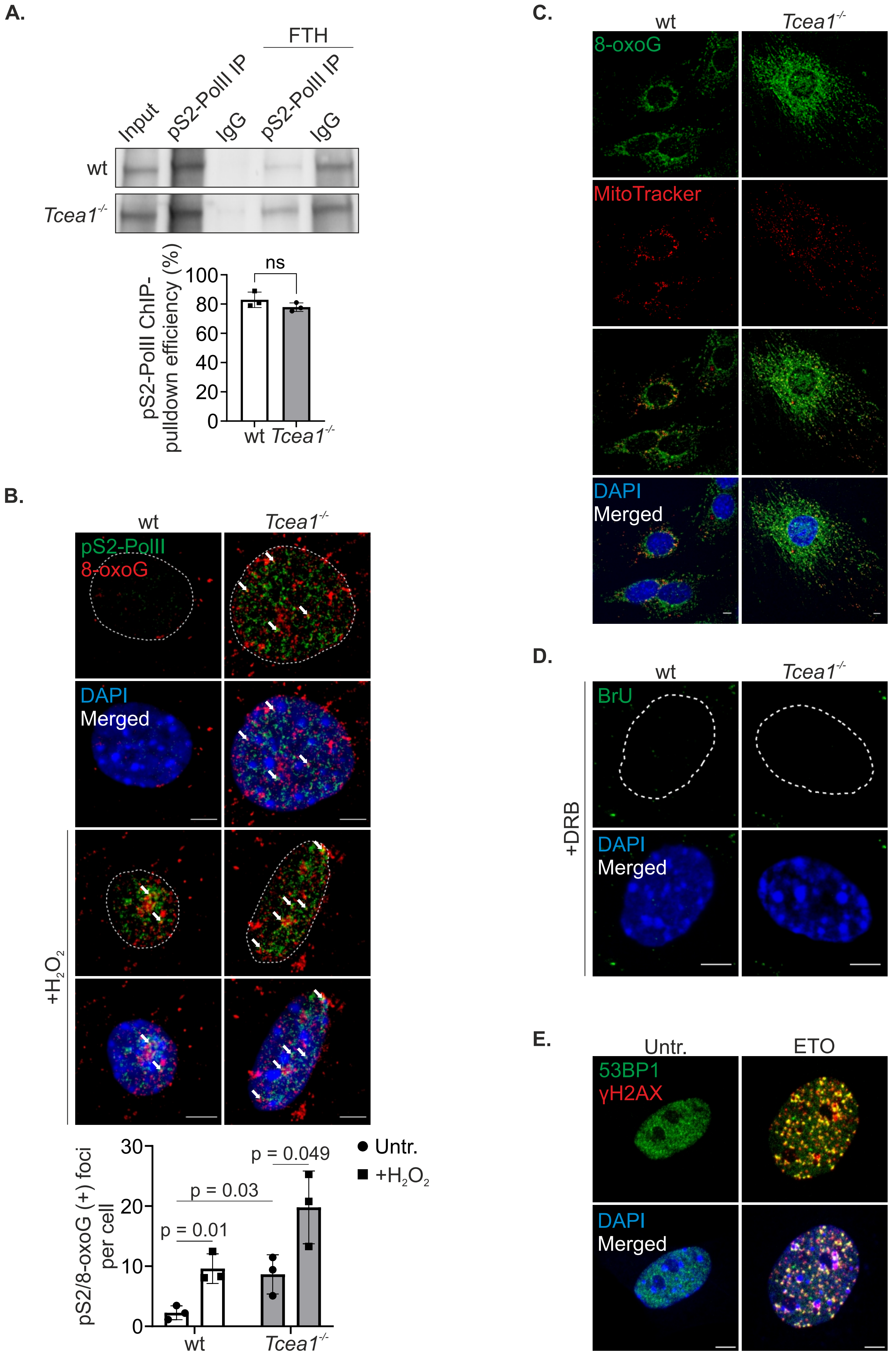

A.
FTH
Input
pS2-PolII IP
IgG
pS2-PolII IP
IgG
wt
Tcea1-/-
pS2-PolII ChIP-pulldown efficiency (%)
ns
100
80
60
40
20
0
wt
Tcea1-/-
B.
wt
Tcea1-/-
pS2-PolII
8-oxoG
DAPI
Merged
+H2O2
pS2/8-oxoG (+) foci per cell
30
20
10
0
p = 0.049
p = 0.03
p = 0.01
Untr.
+H2O2
wt
Tcea1-/-
C.
wt
Tcea1-/-
8-oxoG
MitoTracker
DAPI
Merged
D.
wt
Tcea1-/-
BrU
+DRB
DAPI
Merged
E.
Untr.
ETO
53BP1
γH2AX
DAPI
Merged

Supplementary Figure 4. Oxidative DNA damage-associated transcription stress. (A). Co-immunoprecipitation experiments using anti-pS2-PolII in ChIP-fixed nuclear extracts of wt and *Tcea1*$^{-/-}$ MEFs, analyzed by western blotting for pS2-PolII (n = 3). The graph depicts the pS2-PolII ChIP-pulldown efficiency in wt and *Tcea1*$^{-/-}$ MEFs. **(B)**. Immunofluorescence of 8-oxoG and pS2-PolII in untreated and H_2O_2-treated wt and *Tcea1*$^{-/-}$ MEFs. The graph depicts the percentage of 8-oxoG$^+$; pS2-PolII$^+$ foci per cell (n = 3). **(C)**. 8-oxoG and MitoTracker immunofluorescence in wt and *Tcea1*$^{-/-}$ MEFs. **(D)**. BrU incorporation in DRB-treated wt and *Tcea1*$^{-/-}$ MEFs. **(E).** Immunofluorescence detection of γH2AX and 53BP1 co-localized foci in untreated (Untr.) and etoposide-treated (ETO) wt MEFs. Data analysis was performed using two-tailed Student's *t*-test. All data are presented as mean values ± SEM. Unless otherwise indicated, n = biologically independent experiments and scale bars are set at 5μm. Source data are provided as a Source Data file.

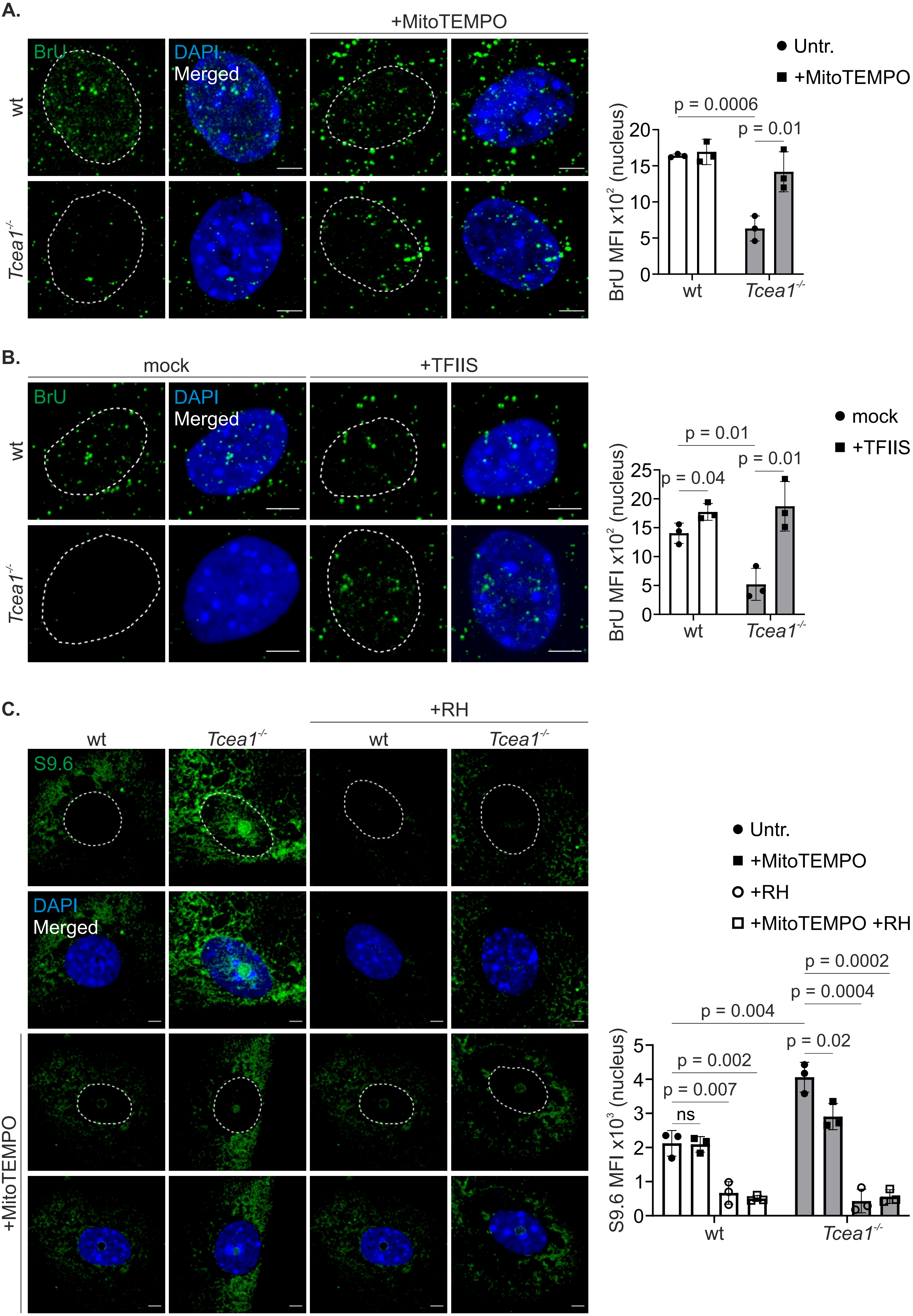
A.
+MitoTEMPO
BrU
DAPI
Merged
wt
Tcea1-/-
Untr.
+MitoTEMPO
p = 0.0006
p = 0.01
BrU MFI x10² (nucleus)
20
15
10
5
0
wt
Tcea1-/-
B.
mock
+TFIIS
BrU
DAPI
Merged
wt
Tcea1-/-
mock
+TFIIS
p = 0.01
p = 0.01
p = 0.04
BrU MFI x10² (nucleus)
25
20
15
10
5
0
wt
Tcea1-/-
C.
+RH
wt
Tcea1-/-
wt
Tcea1-/-
S9.6
DAPI
Merged
+MitoTEMPO
Untr.
+MitoTEMPO
+RH
+MitoTEMPO +RH
p = 0.0002
p = 0.0004
p = 0.004
p = 0.02
p = 0.002
p = 0.007
ns
S9.6 MFI x10³ (nucleus)
5
4
3
2
1
0
wt
Tcea1-/-

Supplementary Figure 5. Transcription stress is ameliorated by antioxidant treatment. (A). BrU incorporation in wt and *Tcea1*$^{-/-}$ MEFs, in the absence or presence of MitoTEMPO. The graph shows the BrU MFI per cell nucleus (n = 3). **(B)**. BrU incorporation in untreated (mock) and TFIIS-overexpressing wt and *Tcea1*$^{-/-}$ MEFs. The graph shows the BrU MFI per cell nucleus (n = 3). **(C)**. Immunostaining using the S9.6 antibody in wt and *Tcea1*$^{-/-}$ MEFs in the absence or presence of MitoTEMPO with/without RNase H treatment. The graph shows the S9.6 MFI per cell nucleus (n = 3). Data analysis was performed using two-tailed Student's *t*-test. All data are presented as mean values ± SEM. Unless otherwise indicated, n = biologically independent experiments and scale bars are set at 5μm. Source data are provided as a Source Data file.

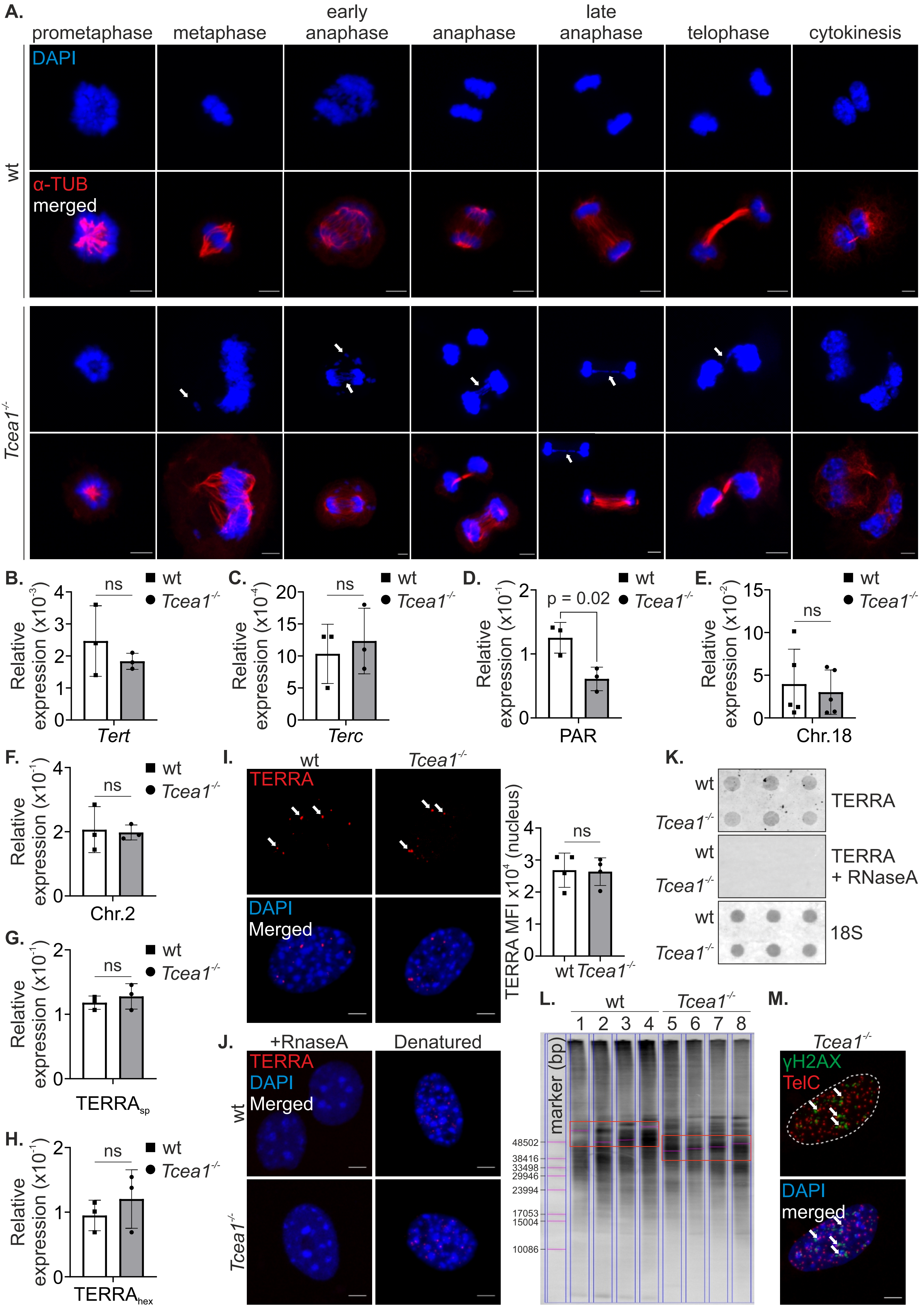

A.
prometaphase
metaphase
early anaphase
anaphase
late anaphase
telophase
cytokinesis
wt
DAPI
α-TUB
merged
Tcea1-/-
B.
Relative expression (x10-3)
ns
wt
Tcea1-/-
Tert
C.
Relative expression (x10-4)
ns
Terc
D.
Relative expression (x10-1)
p = 0.02
PAR
E.
Relative expression (x10-2)
ns
Chr.18
F.
Relative expression (x10-1)
ns
Chr.2
G.
Relative expression (x10-1)
ns
TERRAsp
H.
Relative expression (x10-1)
ns
TERRAhex
I.
wt
Tcea1-/-
TERRA
DAPI
Merged
TERRA MFI x10^4 (nucleus)
ns
J.
+RnaseA
Denatured
TERRA
DAPI
Merged
wt
Tcea1-/-
K.
wt
Tcea1-/-
TERRA
TERRA + RNaseA
18S
L.
wt
Tcea1-/-
1 2 3 4 5 6 7 8
marker (bp)
48502
38416
33498
29946
23994
17053
15004
10086
M.
Tcea1-/-
γH2AX
TelC
DAPI
merged

Supplementary Figure 6. Impaired cell cycle and telomere attrition in *Tcea1*$^{-/-}$ MEFs. (A). Immunofluorescence of cell cycle-synchronized wt and *Tcea1*$^{-/-}$ MEFs. Images show cells during each phase of the cell cycle (arrows depict the presence of DAPI-stained chromatin bridges). *Tert* **(B)**, *Terc* **(C)**, PAR-TERRA **(D)**, Chromosome 18-TERRA (Chr.18) **(E)**, Chromosome 2-TERRA (Chr.2) **(F)**, total TERRA with specific RT primer ($TERRA_{sp}$) **(G)**, and total TERRA with RT with random hexameres ($TERRA_{hex}$) **(H)**, mRNA and RNA levels in wt and *Tcea1*$^{-/-}$ MEFs (n = 3). **(I)**. Fluorescence *in situ* hybridization with $(TTAGGG)_7$-Cy5.5 probe for TERRA in wt and *Tcea1*$^{-/-}$ MEFs. The graph depicts the TERRA MFI per cell nucleus (n = 4). **(J)**. Fluorescence *in situ* hybridization with $(TTAGGG)_7$-Cy5.5 probe for TERRA in wt and *Tcea1*$^{-/-}$ MEFs, treated with RNase A, or under denaturing conditions. **(K)**. TERRA dot blot in untreated and RNase A-treated total RNA samples from wt and *Tcea1*$^{-/-}$ MEFs (n = 3). **(L)**. Southern blot for telomeric length of wt (1-4) and *Tcea1*$^{-/-}$ (5-8) MEFs. Each number represents a biological replicate. **(M).** Immunofluorescence of γH2AX with *in situ* hybridization of telomeric DNA (TelC) in interphase *Tcea1*$^{-/-}$ MEFs. Data analysis was performed using two-tailed Student's *t*-test. All data are presented as mean values ± SEM. Unless otherwise indicated, n = biologically independent experiments and scale bars are set at 5μm. Source data are provided as a Source Data file.

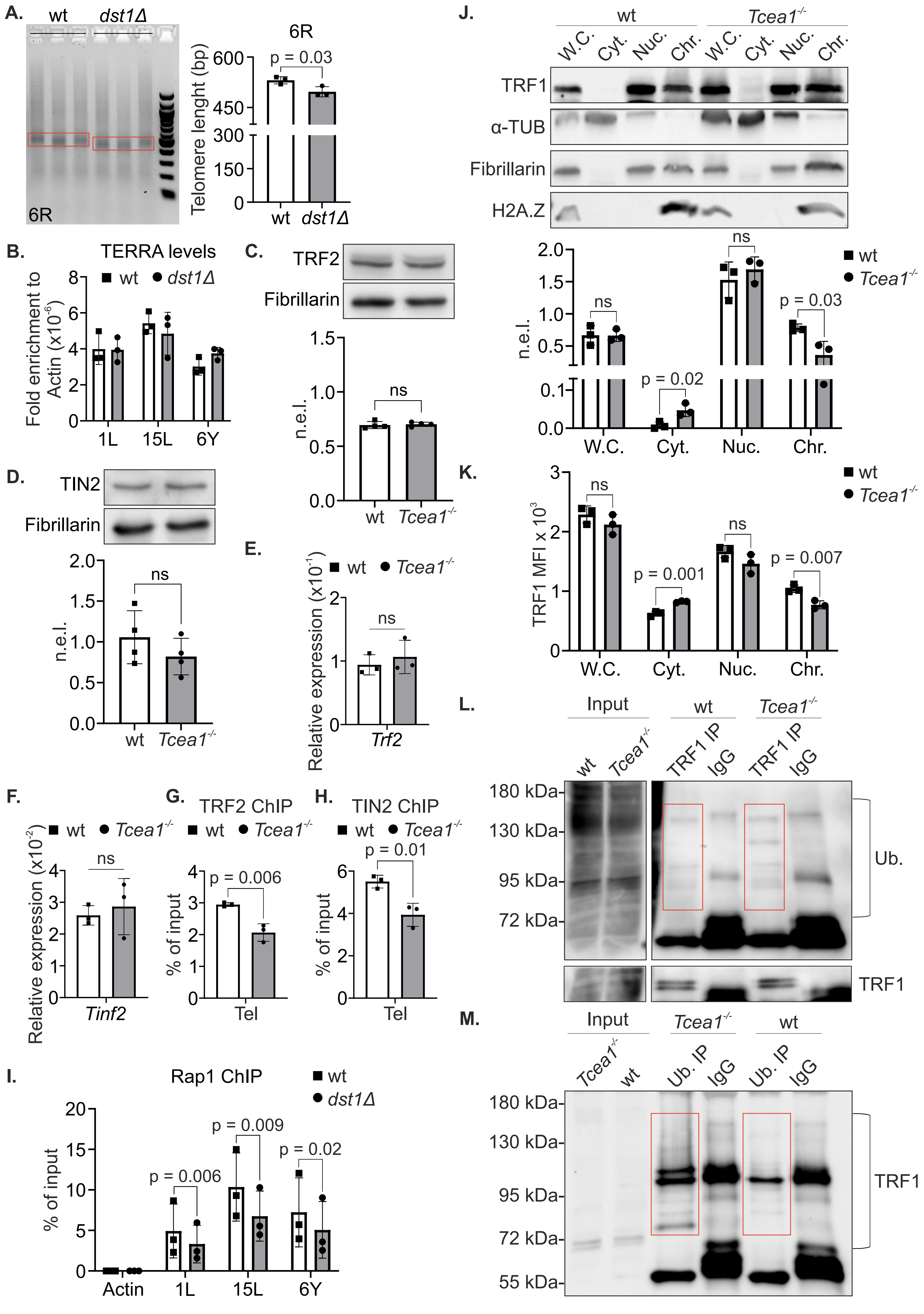

A.
wt
dst1Δ
6R
6R
p = 0.03
Telomere lenght (bp)
600
450
300
150
0
wt
dst1Δ
B.
TERRA levels
wt
dst1Δ
Fold enrichment to Actin (x10^-6)
8
6
4
2
0
1L
15L
6Y
C.
TRF2
Fibrillarin
1.5
1.0
0.5
0.0
n.e.l.
ns
wt
Tcea1-/-
D.
TIN2
Fibrillarin
2.0
1.5
1.0
0.5
0.0
n.e.l.
ns
wt
Tcea1-/-
E.
wt
Tcea1-/-
Relative expression (x10^-1)
2
1
0
ns
Trf2
F.
wt
Tcea1-/-
Relative expression (x10^-2)
ns
4
3
2
1
0
Tinf2
G.
TRF2 ChIP
wt
Tcea1-/-
% of input
p = 0.006
4
3
2
1
0
Tel
H.
TIN2 ChIP
wt
Tcea1-/-
p = 0.01
% of input
6
4
2
0
Tel
I.
Rap1 ChIP
wt
dst1Δ
% of input
20
15
10
5
0
p = 0.006
p = 0.009
p = 0.02
Actin
1L
15L
6Y
J.
wt
Tcea1-/-
W.C.
Cyt.
Nuc.
Chr.
W.C.
Cyt.
Nuc.
Chr.
TRF1
α-TUB
Fibrillarin
H2A.Z
wt
Tcea1-/-
n.e.l.
2.0
1.5
1.0
0.5
0.1
0.0
ns
ns
p = 0.03
p = 0.02
W.C.
Cyt.
Nuc.
Chr.
K.
wt
Tcea1-/-
TRF1 MFI x 10^3
3
2
1
0
ns
ns
p = 0.001
p = 0.007
W.C.
Cyt.
Nuc.
Chr.
L.
Input
wt
Tcea1-/-
wt
Tcea1-/-
TRF1 IP
IgG
TRF1 IP
IgG
180 kDa
130 kDa
95 kDa
72 kDa
Ub.
TRF1
M.
Input
Tcea1-/-
wt
Tcea1-/-
wt
Ub. IP
IgG
Ub. IP
IgG
180 kDa
130 kDa
95 kDa
72 kDa
55 kDa
TRF1

Supplementary Figure 7. Dysfunctional telomeres in *Tcea1*$^{-/-}$ MEFs. (A). Southern blot for telomeric length of *Saccharomyces cerevisiae* chromosome 6R in wt and *dst1Δ* mutant cells. **(B)**. TERRA levels on *Saccharomyces cerevisiae* chromosomes 1L, 15L and 6Y in wt and *dst1Δ* mutant cells. TRF2 **(C)** and TIN2 **(D)** protein levels in whole-cell extracts from wt and *Tcea1*$^{-/-}$ MEFs. Fibrillarin was used to normalize protein expression levels (n.e.l., n = 3). *Trf2* **(E)** and *Tinf2* **(F)** mRNA levels in wt and *Tcea1*$^{-/-}$ MEFs (n = 3). ChIP signals of TRF2 **(G)** and TIN2 **(H)** proteins (shown as percentage of input after IgG normalization) on telomeres of wt and *Tcea1*$^{-/-}$ MEFs (n = 3). **(I)**. ChIP signals of Rap1 protein on telomeres of *Saccharomyces cerevisiae* chromosomes 1L, 15L and 6Y of wt and *dst1Δ* mutant cells. The *Actin* gene was used as a negative control. **(J)**. TRF1 protein levels in whole cell (W.C.), cytoplasmic (Cyt.), nuclear (Nuc.) and chromatin (Chr.) protein extracts from wt and *Tcea1*$^{-/-}$ MEFs (n = 3). α-TUBULIN (α-TUB), Fibrillarin and H2A.Z protein levels were used as loading controls. The graph depicts normalized protein expression levels (n.e.l.) for whole-cell and cytoplasmic protein extracts (α-TUB) and for nuclear and chromatin fractions (Fibrillarin). **(K)**. TRF1 whole cell (W.C), cytoplasmic (Cyt.), nuclear (Nuc.) and chromatin-bound (Chr.) MFI per cell, in wt and Tcea1-/- MEFs, with/without pre-extraction (n=3). Co-immunoprecipitation experiments using anti-TRF1 (TRF1 IP) **(L)** and anti-Ubiquitin (Ub. IP) **(M)** in whole-cell extracts from wt and *Tcea1*$^{-/-}$ MEFs analyzed by western blotting for Ubiquitin and/or TRF1. Red rectangles show the ubiquitin modifications of TRF1 in wt and *Tcea1*$^{-/-}$ MEFs (n = 3). Data analysis was performed using two-tailed Student's *t*-test. All data are presented as mean values ± SEM. Unless otherwise indicated, n = biologically independent experiments and scale bars are set at 5μm. Source data are provided as a Source Data file.

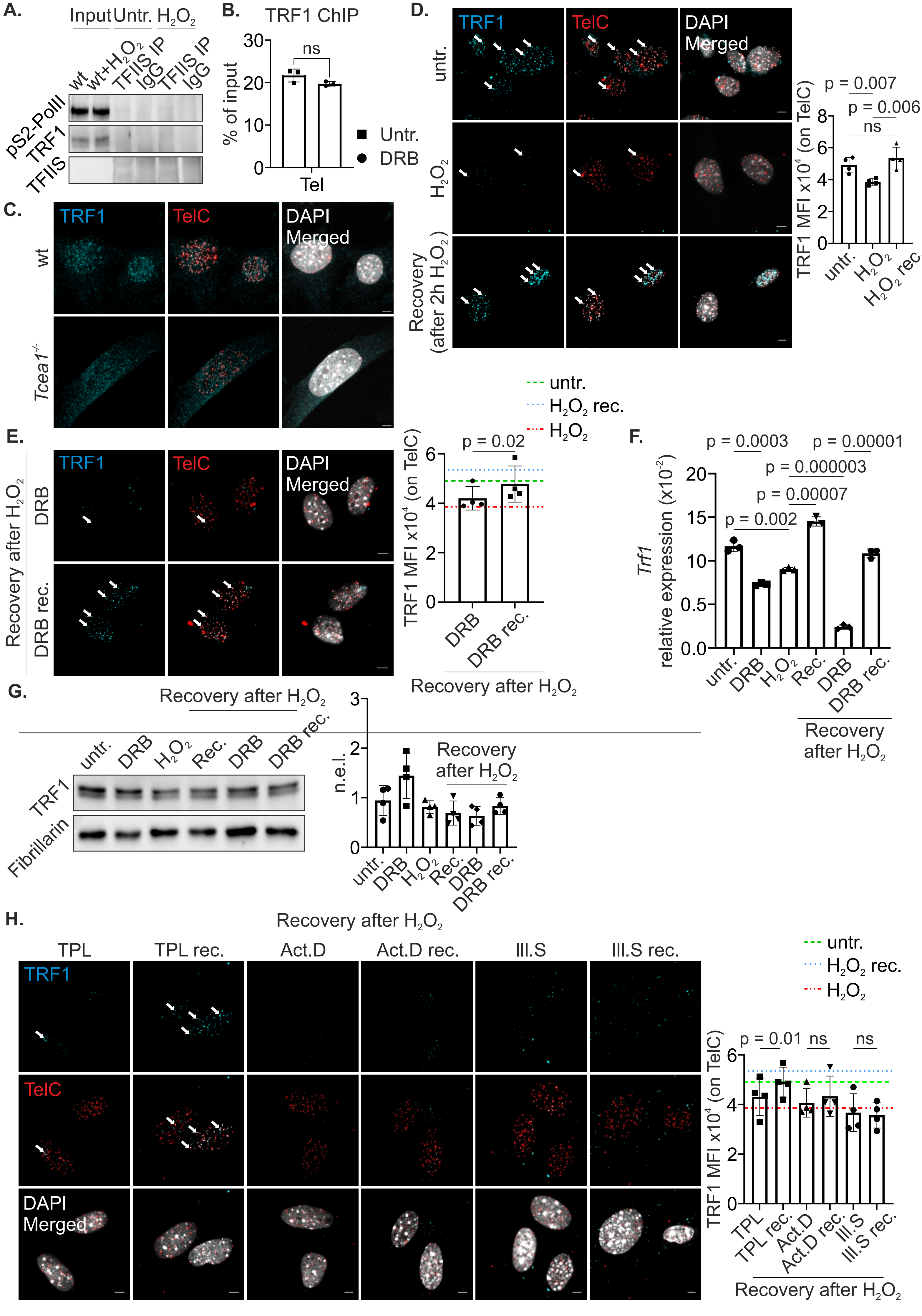

A.
Input
Untr.
H2O2
wt
wt+H2O2
TFIIS IP
IgG
TFIIS IP
IgG
pS2-PolII
TRF1
TFIIS
B.
TRF1 ChIP
ns
% of input
Tel
Untr.
DRB
C.
TRF1
TelC
DAPI Merged
wt
Tcea1-/-
D.
TRF1
TelC
DAPI Merged
untr.
H2O2
Recovery (after 2h H2O2)
p = 0.007
p = 0.006
ns
TRF1 MFI x10^4 (on TelC)
untr.
H2O2
H2O2 rec.
E.
Recovery after H2O2
DRB
DRB rec.
untr.
H2O2 rec.
H2O2
p = 0.02
TRF1 MFI x10^4 (on TelC)
Recovery after H2O2
F.
p = 0.0003
p = 0.00001
p = 0.000003
p = 0.00007
p = 0.002
Trf1 relative expression (x10^-2)
untr.
DRB
H2O2
Rec.
DRB
DRB rec.
Recovery after H2O2
G.
Recovery after H2O2
untr.
DRB
H2O2
Rec.
DRB
DRB rec.
TRF1
Fibrillarin
n.e.l.
Recovery after H2O2
H.
Recovery after H2O2
TPL
TPL rec.
Act.D
Act.D rec.
III.S
III.S rec.
TRF1
TelC
DAPI Merged
untr.
H2O2 rec.
H2O2
p = 0.01
ns
ns
TRF1 MFI x10^4 (on TelC)
Recovery after H2O2

Supplementary Figure 8. Oxidative DNA damage-associated TRF1 release from *Tcea1$^{-/-}$* telomeres. (A). Co-immunoprecipitation experiments using anti-TFIIS in nuclear extracts from *Tcea1$^{-/-}$* MEFs, analyzed by western blotting for pS2-PolII and TRF1 (n = 3). **(B)**. ChIP signals of TRF1 protein (shown as percentage of input after IgG normalization) on telomeres of untreated and DRB-treated wt MEFs (n = 3). **(C)**. Immunofluorescence of TRF1 with *in situ* hybridization of telomeric DNA (TelC) in wt and *Tcea1$^{-/-}$* MEFs. **(D)**. Immunofluorescence of TRF1 with *in situ* hybridization of telomeric DNA (TelC) in wt MEFs (untr: untreated, H_2O_2: treatment with H_2O_2, Rec: H_2O_2-treated, washed and incubated for 16h). White arrows indicate the presence or absence of TRF1 signal on telomeres. The graph shows the TRF1 MFI on telomeric DNA (TelC) in cells (n = 4). **(E)**. Immunofluorescence of TRF1 with *in situ* hybridization of telomeric DNA (TelC) in wt MEFs (DRB: H_2O_2-treated, washed and incubated with DRB for 16h, DRB rec: H_2O_2-treated, washed, incubated with DRB for 16h, washed and incubated for 6h). White arrows indicate the presence or absence of TRF1 signal on telomeres. The graph shows the TRF1 MFI on telomeric DNA (TelC) in cells (green dotted line: untreated, blue dotted line: treatment with H_2O_2, red dotted line: H_2O_2-treated, washed and incubated for 16h, n = 4). **(F)**. *Trf1* mRNA levels in wt MEFs, treated as in (5G) (n = 3). **(G)**. TRF1 protein levels in whole-cell extracts from wt MEFs, treated as in (5G). Fibrillarin was used to normalize protein expression levels (n.e.l., n = 3). **(H)**. Immunofluorescence of TRF1 with *in situ* hybridization of telomeric DNA (TelC) in wt MEFs (TPL/Act.D/III.S: H_2O_2-treated, washed and incubated with TPL/Act.D/III.S for 16h, TPL/Act.D/III.S rec: H_2O_2-treated, washed, incubated with TPL/Act.D/III.S for 16h, washed and incubated for 6h). White arrows indicate the presence or absence of TRF1 signal on telomeres. The graph shows the TRF1 MFI on telomeric DNA (TelC) in cells (green dotted line: untreated, blue dotted line: treatment with H_2O_2, red dotted line: H_2O_2-treated, washed and incubated for 16h, n = 4). Data analysis was performed using two-tailed Student's *t*-test. All data are presented as mean values ± SEM. Unless otherwise indicated, n = biologically independent experiments and scale bars are set at 5μm. Source data are provided as a Source Data file.

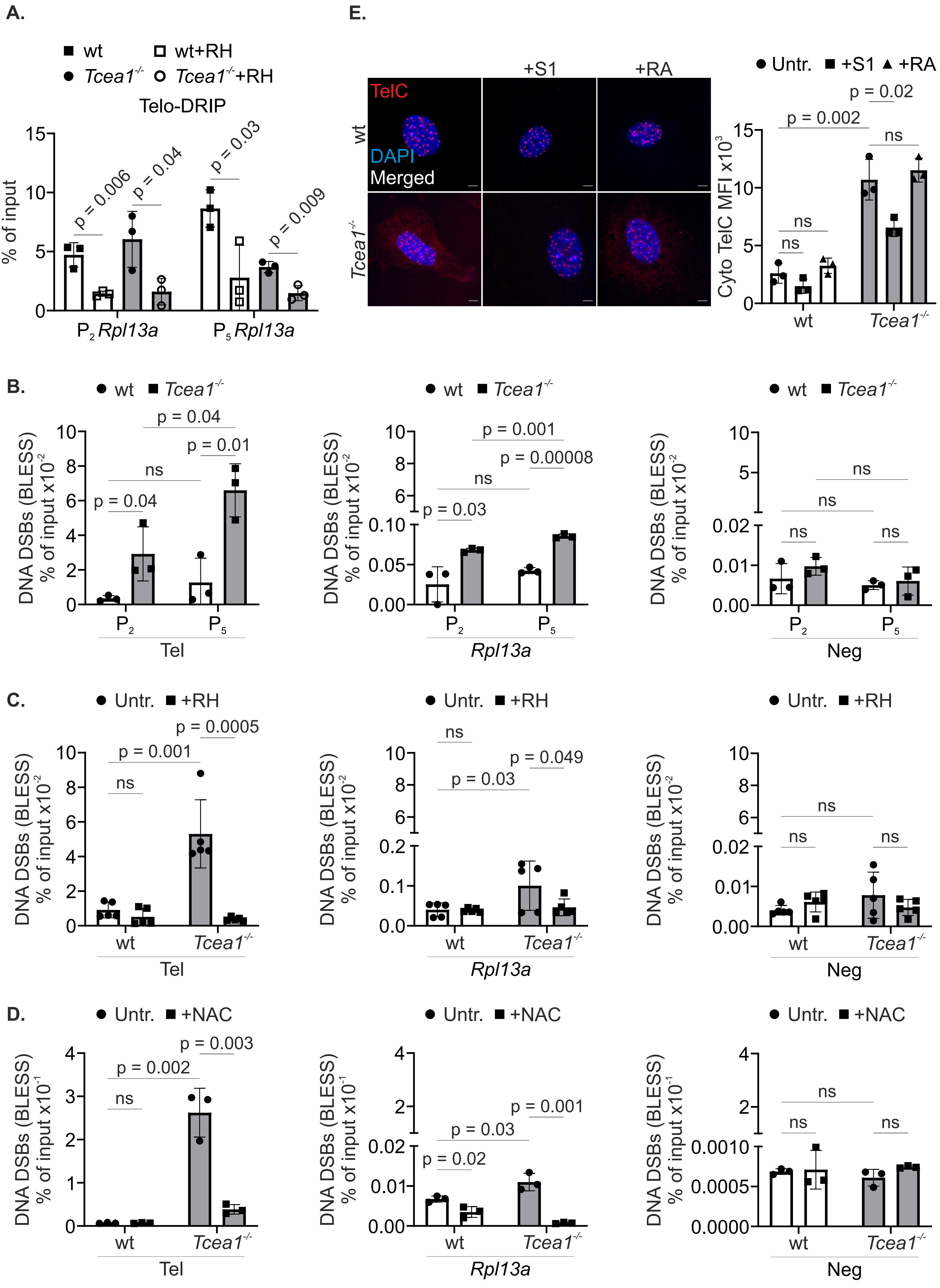

A.
wt
wt+RH
Tcea1-/-
Tcea1-/-+RH
Telo-DRIP
% of input
p = 0.006
p = 0.04
p = 0.03
p = 0.009
P2 Rpl13a
P5 Rpl13a
E.
+S1
+RA
TelC
DAPI
Merged
wt
Tcea1-/-
Untr. +S1 +RA
Cyto TelC MFI x10^3
p = 0.02
p = 0.002
ns
B.
wt Tcea1-/-
DNA DSBs (BLESS) % of input x10^-2
p = 0.04
p = 0.01
p = 0.001
p = 0.00008
p = 0.03
ns
P2
P5
Tel
Rpl13a
Neg
C.
Untr. +RH
p = 0.0005
p = 0.001
p = 0.049
p = 0.03
wt
Tcea1-/-
D.
Untr. +NAC
DNA DSBs (BLESS) % of input x10^-1
p = 0.003
p = 0.002
p = 0.001
p = 0.03
p = 0.02

Supplementary Figure 9. R-loop-induced DNA double strand breaks in *Tcea1*$^{-/-}$ MEFs. (A). DNA-RNA hybrids immunoprecipitation signals using the s9.6 antibody (shown as percentage of input after IgG normalization) on the Rpl13a genetic locus of wt and *Tcea1*$^{-/-}$ MEFs, cultured for two (P_2) or five (P_5) passages (n = 3). BLESS signals quantified by qPCR on telomeres, the Rpl13a gene and a non-transcribed region from Chr.17 (negative; neg.) in wt and *Tcea1*$^{-/-}$ MEFs **(B)** cultured for two (P_2) or five (P_5) passages (n=3), **(C)** untreated (Untr.) or transfected with recombinant RNase H (+RH) (n=5), or **(D)** untreated or treated with the antioxidant NAC (n=3). **(E)**. Fluorescence *in situ* hybridization using a Cy3-PNA TelC probe in either S1 nuclease- (S1) or RNase A- (RA) treated wt and *Tcea1*$^{-/-}$ MEFs. The graph depicts the mean fluorescence intensity (MFI) of TelC in the cytoplasm of cells (n = 3). Data analysis was performed using two-tailed Student's *t*-test. All data are presented as mean values ± SEM. Unless otherwise indicated, n = biologically independent experiments and scale bars are set at 5μm. Source data are provided as a Source Data file.

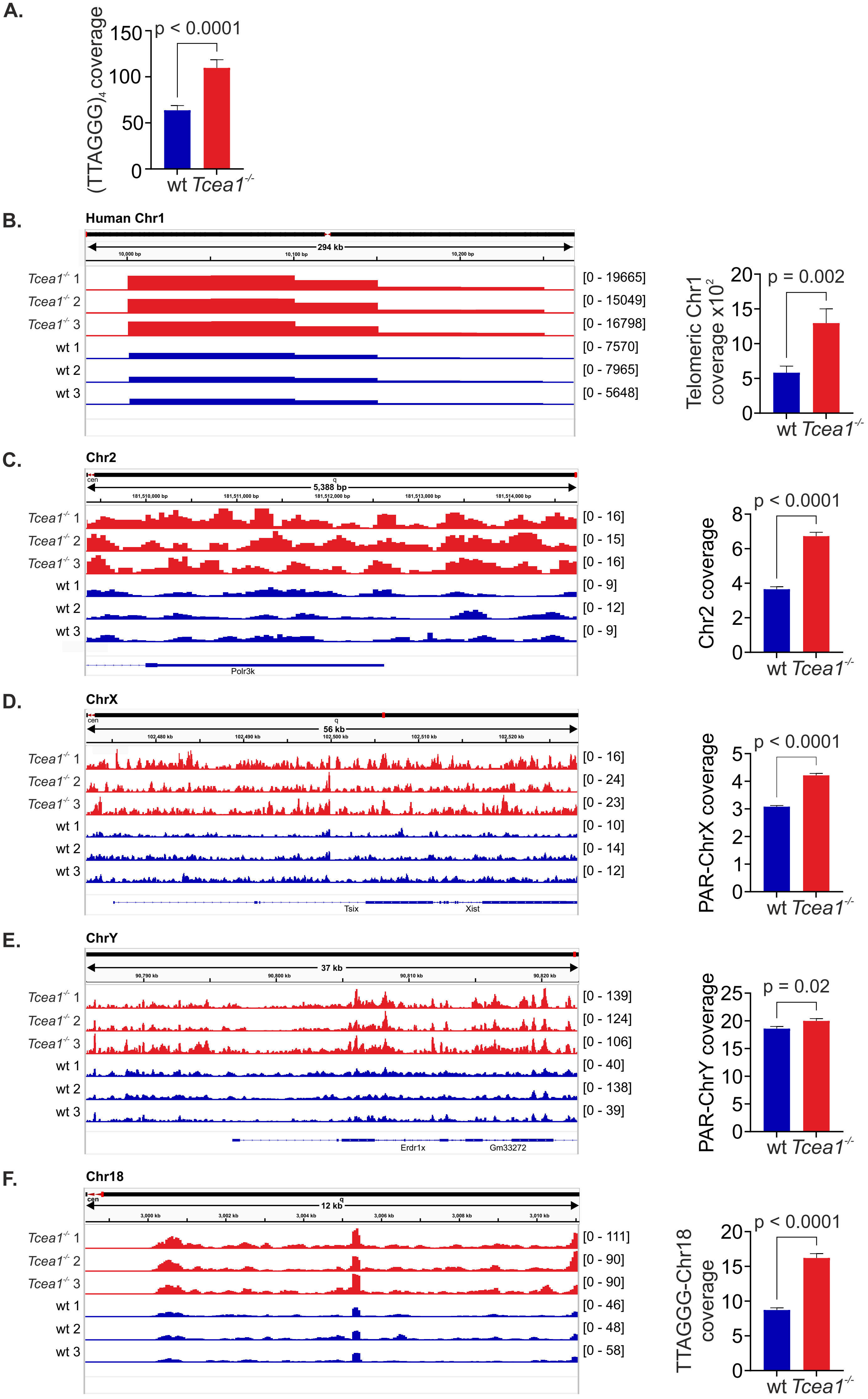
A.
(TTAGGG)4 coverage
p < 0.0001
150
100
50
0
wt Tcea1-/-
B.
Human Chr1
294 kb
Tcea1-/- 1
Tcea1-/- 2
Tcea1-/- 3
wt 1
wt 2
wt 3
[0 - 19665]
[0 - 15049]
[0 - 16798]
[0 - 7570]
[0 - 7965]
[0 - 5648]
Telomeric Chr1 coverage x10^2
p = 0.002
C.
Chr2
5,388 bp
[0 - 16]
[0 - 15]
[0 - 16]
[0 - 9]
[0 - 12]
[0 - 9]
Polr3k
Chr2 coverage
p < 0.0001
D.
ChrX
56 kb
[0 - 16]
[0 - 24]
[0 - 23]
[0 - 10]
[0 - 14]
[0 - 12]
Tsix
Xist
PAR-ChrX coverage
p < 0.0001
E.
ChrY
37 kb
[0 - 139]
[0 - 124]
[0 - 106]
[0 - 40]
[0 - 138]
[0 - 39]
Erdr1x
Gm33272
PAR-ChrY coverage
p = 0.02
F.
Chr18
12 kb
[0 - 111]
[0 - 90]
[0 - 90]
[0 - 46]
[0 - 48]
[0 - 58]
TTAGGG-Chr18 coverage
p < 0.0001

Supplementary Figure 10. Identification of cytoplasmic DNA fragments in *Tcea1$^{-/-}$* MEFs. **(A)**. Quantification of $(TTAGGG)_4$ coverage (sequenced reads/50bp bin) genome-wide, in wt and *Tcea1$^{-/-}$* cytoplasmic DNA (n=3). **(B)**. IGV screenshot showing coverage density of wt and *Tcea1$^{-/-}$* cytoplasmic DNA reads over human Chr.1 telomere. The graph depicts the coverage (sequenced reads/50bp bin) for human Chr.1 telomere. **C-F:** IGV screenshots showing coverage density over indicated regions. The graphs depict the coverage (sequenced reads/50bp bin) for **(C)**. Chr2, **(D)**. ChrX-PAR, **(E)**. ChrY-PAR and **(F)**. Chr18-TTAGGG. Data analysis was performed using two-tailed Student's *t*-test. All data are presented as mean values ± SEM. Unless otherwise indicated, n = biologically independent experiments and scale bars are set at 5μm. Source data are provided as a Source Data file.

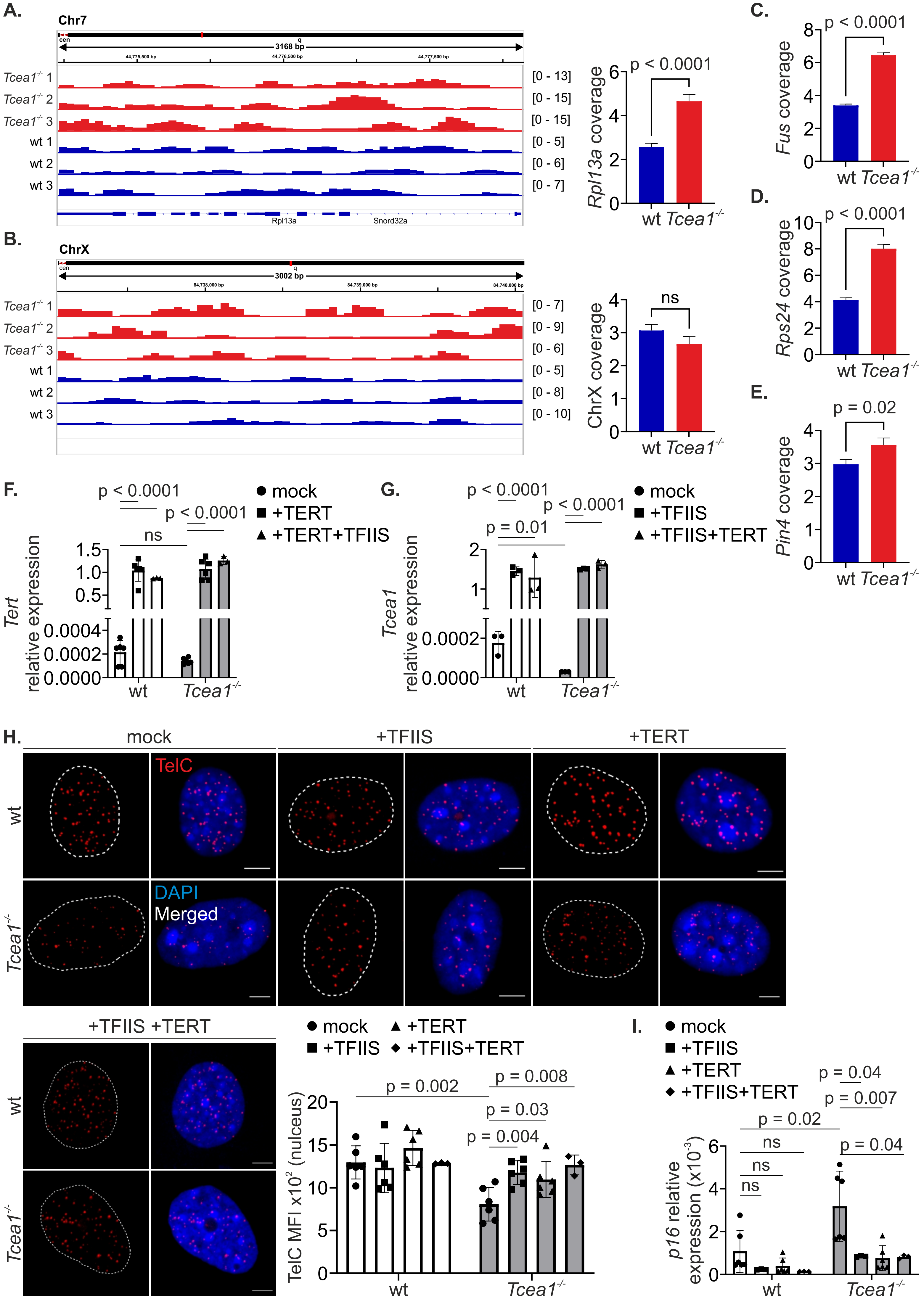
A.
Chr7
3168 bp
Tcea1-/- 1
Tcea1-/- 2
Tcea1-/- 3
wt 1
wt 2
wt 3
[0 - 13]
[0 - 15]
[0 - 15]
[0 - 5]
[0 - 6]
[0 - 7]
Rpl13a
Snord32a
Rpl13a coverage
p < 0.0001
wt Tcea1-/-
B.
ChrX
3002 bp
[0 - 7]
[0 - 9]
[0 - 6]
[0 - 5]
[0 - 8]
[0 - 10]
ChrX coverage
ns
C.
Fus coverage
p < 0.0001
D.
Rps24 coverage
p < 0.0001
E.
Pin4 coverage
p = 0.02
F.
Tert relative expression
p < 0.0001
p < 0.0001
ns
● mock
■ +TERT
▲ +TERT+TFIIS
G.
Tcea1 relative expression
p < 0.0001
p < 0.0001
p = 0.01
● mock
■ +TFIIS
▲ +TFIIS+TERT
H.
mock
+TFIIS
+TERT
TelC
DAPI
Merged
+TFIIS +TERT
● mock ▲ +TERT
■ +TFIIS ◆ +TFIIS+TERT
TelC MFI x10^2 (nulceus)
p = 0.002
p = 0.008
p = 0.03
p = 0.004
I.
● mock
■ +TFIIS
▲ +TERT
◆ +TFIIS+TERT
p16 relative expression (x10^-3)
p = 0.04
p = 0.007
p = 0.02
ns
ns
ns
p = 0.04

Supplementary Figure 11. TERT and TFIIS overexpression ameliorate telomere length in *Tcea1*$^{-/-}$ MEFs. (A). IGV screenshot showing coverage density over *Rpl13a* locus. The graph depicts the coverage (sequenced reads/50bp bin) for *Rpl13a*. **(B)**. IGV screenshot showing coverage density over random ~3kb region (negative control, ChrX). The graph depicts the coverage (sequenced reads/50bp bin) for negative control region. Quantification of *Fus* **(C)**, *Rps24* **(D)** and *Pin4* **(E)** gene regions coverage (sequenced reads/50bp bin) genome-wide, in wt and *Tcea1*$^{-/-}$ cytoplasmic DNA (n=3). **(F)**. *Tert* mRNA levels in untreated (mock), TERT- and TFIIS+TERT-overexpressing wt and *Tcea1*$^{-/-}$ MEFs (n=3). **(G)**. *Tcea1* mRNA levels in untreated (mock), TFIIS- and TFIIS+TERT-overexpressing wt and *Tcea1*$^{-/-}$ MEFs (n=3). **(H)**. Quantitative FISH (Q-FISH) of untreated (mock), TFIIS- ,TERT- and TFIIS+TERT-overexpressing wt and *Tcea1*$^{-/-}$ MEFs using a Cy3-conjugated PNA probe for telomeric DNA (n = 3). The graph depicts the mean fluorescence intensity (MFI) per cell nucleus. **(I)**. *p16* mRNA levels in untreated (mock), TFIIS-, TERT- and TFIIS+TERT-overexpressing wt and *Tcea1*$^{-/-}$ MEFs (n=3). Data analysis was performed using two-tailed Student's *t*-test. All data are presented as mean values ± SEM. Unless otherwise indicated, n = biologically independent experiments and scale bars are set at 5μm. Source data are provided as a Source Data file.

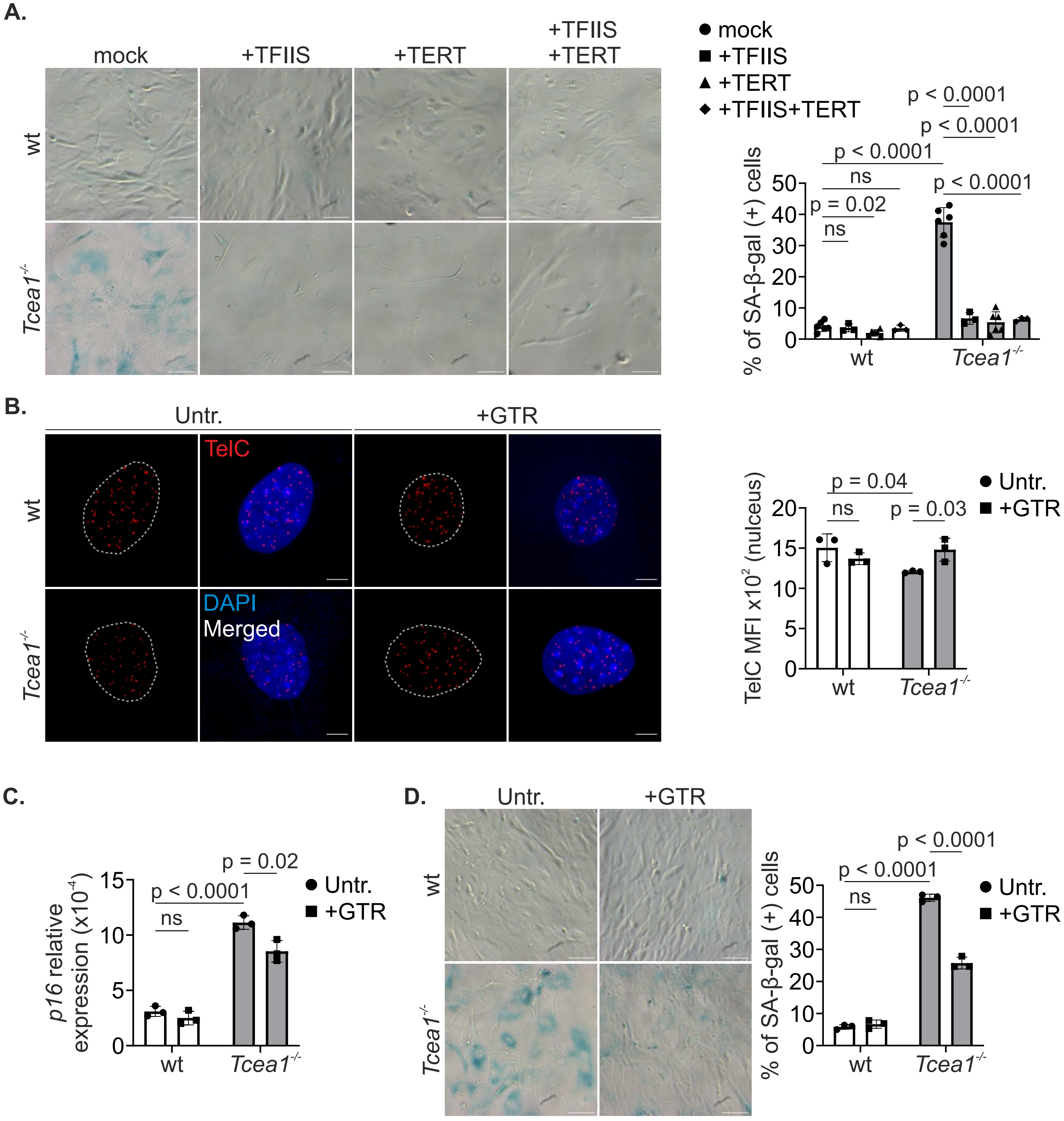
A.
mock
+TFIIS
+TERT
+TFIIS
+TERT
wt
Tcea1-/-
mock
+TFIIS
+TERT
+TFIIS+TERT
p < 0.0001
p < 0.0001
p < 0.0001
ns
p = 0.02
ns
p < 0.0001
% of SA-β-gal (+) cells
50
40
30
20
10
0
wt
Tcea1-/-
B.
Untr.
+GTR
TelC
DAPI
Merged
wt
Tcea1-/-
p = 0.04
ns
p = 0.03
Untr.
+GTR
TelC MFI x10² (nulceus)
20
15
10
5
0
wt
Tcea1-/-
C.
p = 0.02
p < 0.0001
ns
Untr.
+GTR
p16 relative expression (x10⁻⁴)
15
10
5
0
wt
Tcea1-/-
D.
Untr.
+GTR
wt
Tcea1-/-
p < 0.0001
p < 0.0001
ns
Untr.
+GTR
% of SA-β-gal (+) cells
50
40
30
20
10
0
wt
Tcea1-/-

Supplementary Figure 12. Telomere extension ameliorates cellular senescence in *Tcea1*$^{-/-}$ MEFs. (A). SA-β-gal assay in untreated (mock), TFIIS-, TERT- and TFIIS+TERT-overexpressing wt and *Tcea1*$^{-/-}$ MEFs. The graph depicts the % of SA-β-gal-positive (+) cells (n = 3). Scale bar is set at 20μm. **(B)**. Quantitative FISH (Q-FISH) of untreated and GTR-treated (G-rich terminal repeats) wt and *Tcea1*$^{-/-}$ MEFs using a Cy3-conjugated PNA probe for telomeric DNA (n = 3). The graph depicts the mean fluorescence intensity (MFI) per cell nucleus. **(C)**. *p16* mRNA levels in untreated and GTR-treated (G-rich terminal repeats) wt and *Tcea1*$^{-/-}$ MEFs (n=3). **(D)**. SA-β-gal assay in untreated and GTR-treated (G-rich terminal repeats) wt and *Tcea1*$^{-/-}$ MEFs. The graph depicts the % of SA-β-gal-positive (+) cells (n = 3). Scale bar is set at 20μm. Data analysis was performed using two-tailed Student's *t*-test. All data are presented as mean values ± SEM. Unless otherwise indicated, n = biologically independent experiments and scale bars are set at 5μm. Source data are provided as a Source Data file.

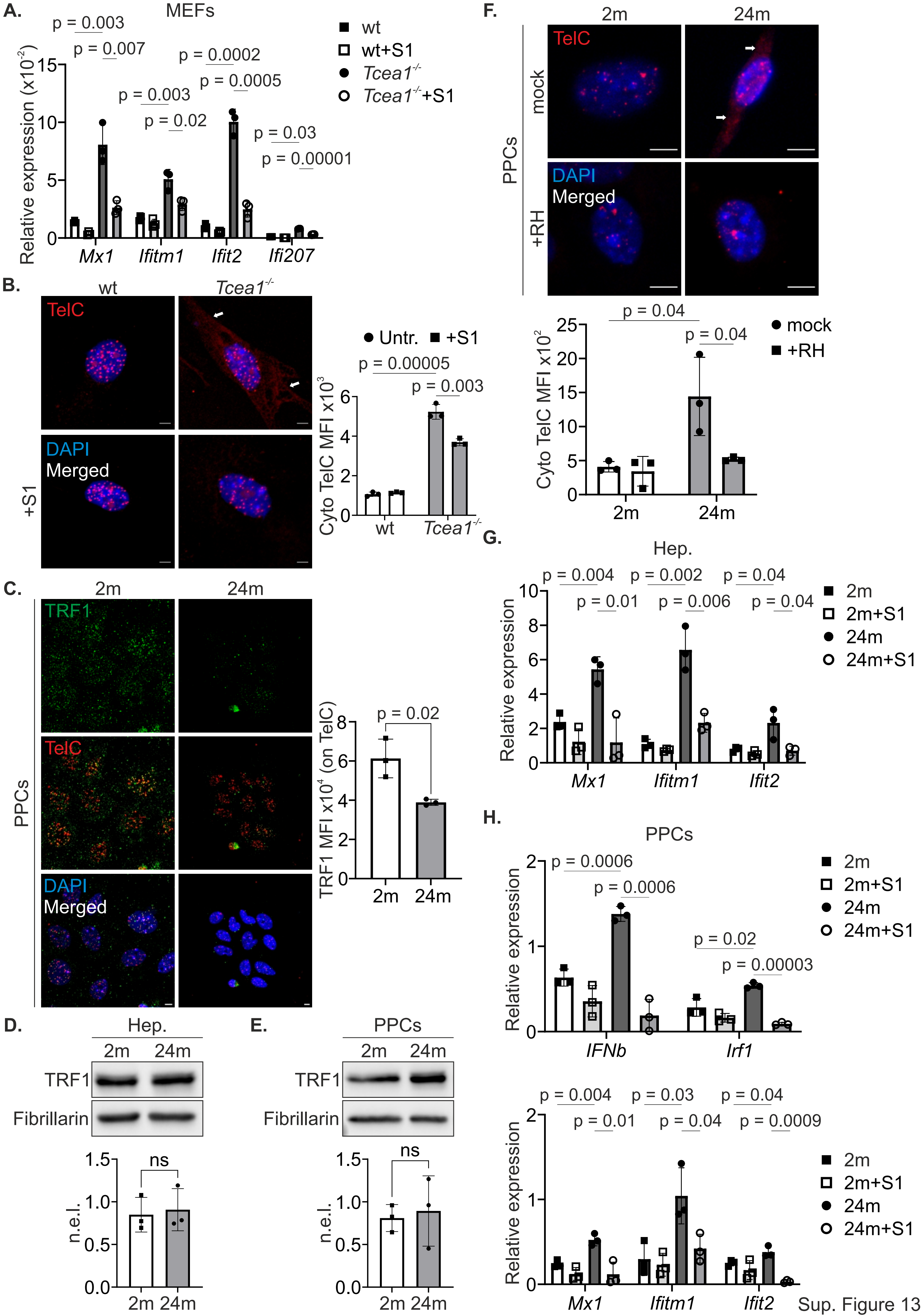

Sup. Figure 13

Supplementary Figure 13. R-loop-derived cytosolic telomeric fragments in *Tcea1*$^{-/-}$ PPCs. **(A)**. *Mx1*, *Ifitm1*, *Ifit2*, *and Ifi207* mRNA levels from wt and *Tcea1*$^{-/-}$ MEFs, untreated or incubated with NIH-derived, S1 nuclease-loaded vesicles (n = 3). **(B)**. Fluorescence *in situ* hybridization using a Cy3-PNA TelC probe in wt and *Tcea1*$^{-/-}$ MEFs, untreated or incubated with NIH-derived, S1 nuclease-loaded vesicles. The graph depicts the mean fluorescence intensity (MFI) of TelC in the cytoplasm of cells (n = 3). **(C)**. Immunofluorescence of TRF1 with *in situ* hybridization of telomeric DNA (TelC) in primary pancreatic cells (PPCs) from 2-month- and 24-month-old mice. The graph depicts the TRF1 MFI on telomeres of pancreatic cells (n = 3). TRF1 protein levels in whole-cell extracts from primary hepatocytes **(D)** and pancreatic **(E)** cells. Fibrillarin was used to normalize protein expression levels (n.e.l., n = 3). **(F)**. Fluorescence *in situ* hybridization using a Cy3-PNA TelC probe in primary pancreatic cells from 2-month- and 24-month-old mice, either untreated or transfected with RNase H (RH). The graph depicts the mean fluorescence intensity (MFI) of TelC in the cytoplasm of pancreatic cells (n = 3). **(G)**. *Mx1*, *Ifitm1* and *Ifit2* mRNA levels in hepatocytes from 2-month and 24-month-old mice, untreated or incubated with NIH-derived, S1 nuclease-loaded vesicles (n ≥ 3). **(H)**. *Ifnβ, Mx1*, *Ifitm1*, *Ifit2* and *Irf1* mRNA levels in primary pancreatic cells from 2-month and 24-month-old mice, untreated or incubated with NIH-derived, S1 nuclease-loaded vesicles (n = 3). Data analysis was performed using two-tailed Student's *t*-test. All data are presented as mean values ± SEM. Unless otherwise indicated, n = biologically independent experiments and scale bars are set at 5µm. Source data are provided as a Source Data file.

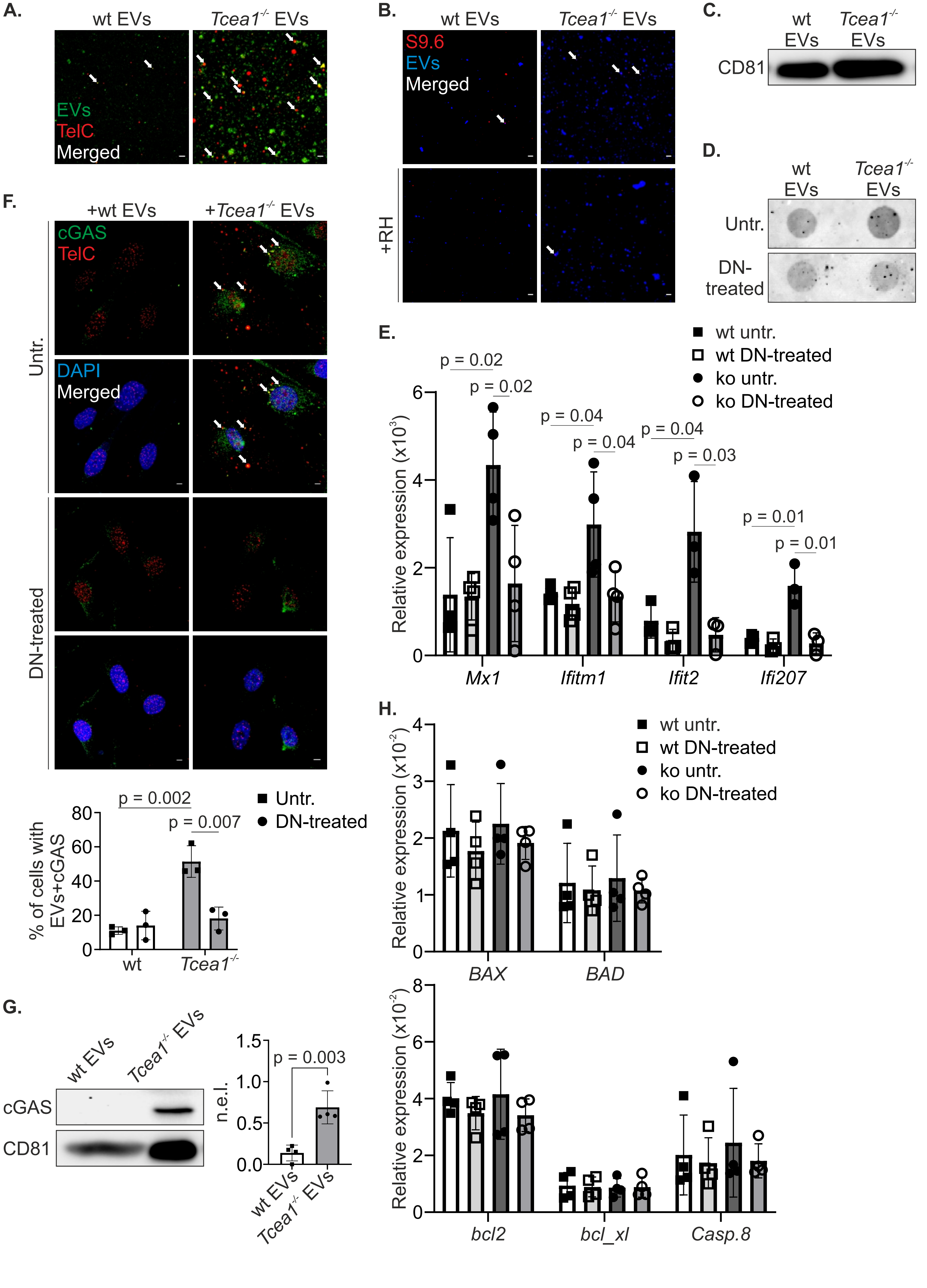

Sup. Figure 14

Supplementary Figure 14. ***Tcea1**$^{-/-}$* **MEFs secrete TelC-loaded EVs.** **(A)**. Representative images of ExoFlow-stained EVs from wt and *Tcea1*$^{-/-}$ MEFs, with *in situ* hybridization of telomeric DNA (TelC). Scale bar is set at 1µm. **(B)**. Representative images of ExoFlow-stained EVs from wt and *Tcea1*$^{-/-}$ MEFs, with immunostaining for S9.6, in the absence or presence of RNase H. Scale bar is set at 1µm. **(C)**. Western blotting of CD81 protein in EVs from $5x10^6$ wt and *Tcea1*$^{-/-}$ MEFs (n = 3). **(D)**. Dot blot using an Alexa488-PNA TelC probe in untreated or DNase I-treated (DN-treated) EVs, isolated from wt and *Tcea1*$^{-/-}$ MEFs. **(E)**. *Mx1*, *Ifitm1*, *Ifit2 and Ifi207* mRNA levels in wt MEFs incubated with untreated or DNase I-treated EVs from wt and *Tcea1*$^{-/-}$ MEFs (n ≥ 3). **(F)**. Immunofluorescence of cGAS with *in situ* hybridization of telomeric DNA (TelC) in wt MEFs incubated with untreated or DNase I-treated EVs from wt and *Tcea1*$^{-/-}$ MEFs. The graph depicts the percentage of cells with $cGAS^+$;$TelC^+$ EVs (n = 3). **(G)**. cGAS protein levels in EVs from $5x10^6$ wt and *Tcea1*$^{-/-}$ MEFs. CD81 was used to normalize cGAS levels (n.e.I., n = 3). **(H)**. *Bcl2*, *Bcl_xl*, *Casp.8*, *BAX*, and *BAD* mRNA levels in wt MEFs incubated with untreated or DNase I-treated EVs from wt and *Tcea1*$^{-/-}$ MEFs (red dashed line: untreated wt EVs, n = 4). Data analysis was performed using two-tailed Student's *t*-test. All data are presented as mean values ± SEM. Unless otherwise indicated, n = biologically independent experiments and scale bars are set at 5µm. Source data are provided as a Source Data file.

Supplementary Table 1. Yeast strains

Strains	Mating type	Background	Free plasmid	Genotype	Description
yFB1424	a	S288C	pBL906	*MAT* **a** *his3Δ1 leu2Δ0 met15Δ0 ura3Δ0*	Wild type (BY4741) transformed with EV (pRS416-GAL)
yFB1428	a	S288C	pBL837	*MAT* **a** *his3Δ1 leu2Δ0 met15Δ0 ura3Δ0*	Wild type (BY4741) transformed with H1(D193N) (pRS416-GAL)
yFB1743	a	S288C	pBL906	*MAT* **a** *his3Δ1 leu2Δ0 met15Δ0 ura3Δ0 dst1::KAN*	*dst1* (yFB1739) transformed with EV (pRS416-GAL)
yFB1749	a	S288C	pBL837	*MAT* **a** *his3Δ1 leu2Δ0 met15Δ0 ura3Δ0 dst1::KAN*	*dst1* (yFB1739) transformed with H1(D193N) (pRS416-GAL)
