# Supplementary material for: Transcription stress at telomeres leads to cytosolic DNA release and paracrine senescence
Source: Nat Commun. 2024 May 14;15:4061. doi: 10.1038/s41467-024-48443-6 (PMC11094137; doi:10.1038/s41467-024-48443-6)
Supplement: Supplementary file 3 — Description of Additional Supplementary Files [file 41467_2024_48443_MOESM3_ESM.pdf]

Description of Additional Supplementary Files

File Name: Supplementary Data 1
Description: Oligonucleotides used in PCR, qPCR and FISH/dot-blots.
