# Supplementary material for: Transcription stress at telomeres leads to cytosolic DNA release and paracrine senescence
Source: Nat Commun. 2024 May 14;15:4061. doi: 10.1038/s41467-024-48443-6 (PMC11094137; doi:10.1038/s41467-024-48443-6)
Supplement: Supplementary file 5 — Reporting Summary [file 41467_2024_48443_MOESM5_ESM.pdf]

Corresponding author(s): George A. Garinis
Last updated by author(s): Apr 5, 2024

Reporting Summary

Nature Portfolio wishes to improve the reproducibility of the work that we publish. This form provides structure for consistency and transparency in reporting. For further information on Nature Portfolio policies, see our Editorial Policies and the Editorial Policy Checklist.

Statistics

For all statistical analyses, confirm that the following items are present in the figure legend, table legend, main text, or Methods section.

n/a	Confirmed	
☐	☒	The exact sample size (*n*) for each experimental group/condition, given as a discrete number and unit of measurement
☐	☒	A statement on whether measurements were taken from distinct samples or whether the same sample was measured repeatedly
☐	☒	The statistical test(s) used AND whether they are one- or two-sided<br>*Only common tests should be described solely by name; describe more complex techniques in the Methods section.*
☐	☐	A description of all covariates tested
☐	☐	A description of any assumptions or corrections, such as tests of normality and adjustment for multiple comparisons
☐	☒	A full description of the statistical parameters including central tendency (e.g. means) or other basic estimates (e.g. regression coefficient) AND variation (e.g. standard deviation) or associated estimates of uncertainty (e.g. confidence intervals)
☐	☒	For null hypothesis testing, the test statistic (e.g. *F*, *t*, *r*) with confidence intervals, effect sizes, degrees of freedom and *P* value noted<br>*Give P values as exact values whenever suitable.*
☒	☐	For Bayesian analysis, information on the choice of priors and Markov chain Monte Carlo settings
☒	☐	For hierarchical and complex designs, identification of the appropriate level for tests and full reporting of outcomes
☒	☐	Estimates of effect sizes (e.g. Cohen's *d*, Pearson's *r*), indicating how they were calculated

Our web collection on statistics for biologists contains articles on many of the points above.

Software and code

Policy information about availability of computer code

Data collection	N/A. This paper does not report original code.
Data analysis	ImageJ 1.53q, GraphPad Prism 9.0.0 (121), STAR aligner 2.7.11b, edgeR 4.0.16, DESeq 1.12.1, NBPSeq 0.3.1, metaseqR2 1.1.23, FastQC v0.12.1, TrimmomaticPE 0.39, IGV 2.4.14. This paper does not report original code.

For manuscripts utilizing custom algorithms or software that are central to the research but not yet described in published literature, software must be made available to editors and reviewers. We strongly encourage code deposition in a community repository (e.g. GitHub). See the Nature Portfolio guidelines for submitting code & software for further information.

Data

Policy information about availability of data

All manuscripts must include a data availability statement. This statement should provide the following information, where applicable:

- Accession codes, unique identifiers, or web links for publicly available datasets
- A description of any restrictions on data availability
- For clinical datasets or third party data, please ensure that the statement adheres to our policy

All data generated during this study are included in the published article (and its supplementary information files). The RNA-Seq data (PRJEB63556) are deposited in ArrayExpress (https://www.ebi.ac.uk/arrayexpress/). The Cytoseq data (PRJEB71474) are deposited on the European Nucleotide Archive. Information and requests for resources and reagents should be directed to and will be fulfilled by the lead contact, George A. Garinis (garinis@imbb.forth.gr). Original western blot images, microscopy data and all other raw data will be shared by the lead contact upon request.

Research involving human participants, their data, or biological material

Policy information about studies with human participants or human data. See also policy information about sex, gender (identity/presentation), and sexual orientation and race, ethnicity and racism.

Reporting on sex and gender	N/A
Reporting on race, ethnicity, or other socially relevant groupings	N/A
Population characteristics	N/A
Recruitment	N/A
Ethics oversight	N/A

Note that full information on the approval of the study protocol must also be provided in the manuscript.

Field-specific reporting

Please select the one below that is the best fit for your research. If you are not sure, read the appropriate sections before making your selection.

☒ Life sciences ☐ Behavioural & social sciences ☐ Ecological, evolutionary & environmental sciences

For a reference copy of the document with all sections, see nature.com/documents/nr-reporting-summary-flat.pdf

Life sciences study design

All studies must disclose on these points even when the disclosure is negative.

Sample size	Sample size calculation was based on 1. effect size (the difference between the mean of two groups), 2. the standard deviation or standard error of the mean variability within the sample, 3. the decision of direction of effect (two tailed for all experiments performed). According to these criteria n was calculated to be the minimum required number of experiments (least possible animals used) to provide the study with reasonable statistical power.
Data exclusions	No data were excluded from the analysis.
Replication	All experiments were reproduced at least 3 times (biological replicates) and in multiple technical replicates (at least 3 technical replicates).
Randomization	Samples were allocated to experimental groups according to genotypes or treatments.
Blinding	Investigators were not blinded to group allocations for all experiments performed in this work. This is due to the fact that the cells used in this work have obvious phenotypes.

Reporting for specific materials, systems and methods

We require information from authors about some types of materials, experimental systems and methods used in many studies. Here, indicate whether each material, system or method listed is relevant to your study. If you are not sure if a list item applies to your research, read the appropriate section before selecting a response.

Materials & experimental systems

n/a	Involved in the study
☐	☒ Antibodies
☒	☐ Eukaryotic cell lines
☒	☐ Palaeontology and archaeology
☐	☒ Animals and other organisms
☒	☐ Clinical data
☒	☐ Dual use research of concern
☒	☐ Plants

Methods

n/a	Involved in the study
☒	☐ ChIP-seq
☐	☒ Flow cytometry
☒	☐ MRI-based neuroimaging

Antibodies

Antibodies used	Antibodies against γH2AX (05-636, IF: 1:12000), s9.6 (MABE1095, IF: 1:100, Telo-DRIP: 5μg), 8-oxoG (MAB3560, IF: 1:100, WB: 1:1000, oxi-DIP: 4μg), goat anti-rabbit IgG-HRP (AP132P, WB: 1:10000), goat anti-mouse IgG-HRP (AP124P, WB: 1:5000) and mouse IgM negative control (MABC008) were from Millipore. Antibodies against γH2AX (ab22551, WB: 1:1000), fibrillarin (ab5821, WB: 1:2500), TRF1 (ab192629, IF: 1:100), β-tubulin (ab6046, WB: 1:5000), TFIIS (ab185947, WB: 1:1000, IP: 6μg), TRF1 (ab10579, WB:1:500, IP/ChIP: 6μg) and pS2-PolII (ab5095, IF: 1:1000, WB: 1:1000, ChIP: 6μg) were from Abcam. Antibodies against cleaved caspase 3 (9661, IF: 1:50, WB: 1:500), pATM (4526, IF: 1:100), α-tubulin (3873, IF: 1:2000), cGAS (31659, IF: 1:100, WB: 1:1000), CD81 (10037, WB: 1000) and H2A.Z (2718, WB: 1:1000) were from Cell Signaling Technology. Antibodies against 53BP1 (NB100-304, IF: 1:200), ATM (NB100-220, WB: 1:500) and goat anti-mouse IgM 550 (NB120-9167R, IF: 1:200) were from Novus Biologicals. Anti-BrdU antibody (555627, IF: 1:250, FACS: 1μg/10^6 cells) was from BD Pharmingen. Antibodies against TOM20 (sc17764, IF: 1:50), RNAPII (sc-55492, WB: 1:500), Ubiquitin (sc-8017, WB: 1:1000, IP: 6μg), yeast Rap1 (sc-20167, ChIP: 5μg) and normal mouse IgG (sc-2025) were form Santa Cruz. Antibodies against TRF1 (67592-1-Ig, WB: 1:500, IP/ChIP: 6μg), TRF2 (66893-1-Ig, WB:1:500, ChIP: 6μg) and TIN2 (11368-1-AP, WB:1:500, ChIP: 6μg) were from Proteintech. Antibody against pATM (200-301-400, WB: 1:300) was from Rockland. IgG from rabbit serum (I5006) was from Sigma. Goat anti-mouse IgG Alexa Fluor 488 (A-11001, IF: 1:2000, FACS: 1:250), goat anti-mouse IgG AlexaFluor 555 (A-21422, IF: 1:2000), donkey anti-rabbit IgG AlexaFluor 488 (A-21206, IF: 1:2000), goat anti-rat IgG AlexaFluor 647 (A-21247, IF: 1:2000), donkey anti-rabbit IgG AlexaFluor 555 (A-31572, IF: 1:2000) and DAPI (62247, IF: 1:20000) were from ThermoFisher Scientific. For the SA-β-gal activity, the Beta-galactosidase (β-gal) assay kit was used (9860, Cell Signaling Technology) according to the manufacturer's instructions.
Validation	All antibodies used in this study were validated by the manufacturer.

Animals and other research organisms

Policy information about studies involving animals; ARRIVE guidelines recommended for reporting animal research, and Sex and Gender in Research

Laboratory animals	Mus musculus; strain: C57BL/6, Tcea1 fl/fl, CMV-Cre; sex: males or females; age: 2-month old and 24-month old mice.
Wild animals	There was no use of wild animals.
Reporting on sex	There are no sex-dependent data. Sex was not considered in this study design.
Field-collected samples	There is no work with field-collected samples.
Ethics oversight	Animals were kept on a regular diet and housed at the IMBB animal house, which operates in compliance with the "Animal Welfare Act" of the Greek government, using the "Guide for the Care and Use of Laboratory Animals" as its standard. As required by Greek law, formal permission to generate and use genetically modified animals was obtained from the responsible local and national authorities. All animal studies were approved by independent Animal Ethical Committees at FORTH.

Note that full information on the approval of the study protocol must also be provided in the manuscript.

Plants

Seed stocks	N/A
Novel plant genotypes	N/A
Authentication	N/A

Flow Cytometry

Plots

Confirm that:

- [x] The axis labels state the marker and fluorochrome used (e.g. CD4-FITC).
- [x] The axis scales are clearly visible. Include numbers along axes only for bottom left plot of group (a 'group' is an analysis of identical markers).
- [x] All plots are contour plots with outliers or pseudocolor plots.
- [x] A numerical value for number of cells or percentage (with statistics) is provided.

Methodology

Sample preparation	For cell cycle analysis cells were fixed with 70% ethanol for at least 1h and incubated with 2N HCl/0.5% Triton-100 for 30min, RT. Cells were then resuspended in 0.1M sodium tetraborate for 2min, washed with PBS/1% BSA and incubated with Anti-BrdU in 0.5% Tween 20/1% BSA/PBS for 1h, RT. After washing with PBS/1% BSA, cells were stained with anti-mouse IgG 488 for 30min, RT and then incubated with RNase A (10µg/ml) and propidium iodide (20µg/ml) for 30min, RT. The FITC Annexin V Apoptosis Detection Kit I (556547, BD Biosciences) was used for Annexin V – Propidium Iodide staining according to the manufacturer's instructions. For proliferation assay cells were stained with CellTrace CFSE Cell Proliferation Kit, for flow cytometry (C34554, Invitrogen) according to the manufacturer's instructions.
Instrument	FACS Calibur (BD Biosciences)
Software	We collected and analyzed the data using FlowJo 10.5.3 software.
Cell population abundance	No sorting was performed with the flow-cytometer.
Gating strategy	To gate samples for FACS analysis, cells were initially gated by Forward Scatter VS Side Scatter to separate cell events from debris. For Annexin V-PI analysis, a gate was set to evaluate the numbers of Annexin V(-)-PI(-), Annexin V(+)-PI(-), Annexin V (-)-PI(+) and Annexin V(+)-PI(+) populations. For cell proliferation analysis, gates were set using the proliferation modeling tool from FlowJo. In both cases, fluorescence pattern of unstained cells (background fluorescence) and single stained cells (for cell cycle assay) were used to set the parameters of the gates.

- [ ] Tick this box to confirm that a figure exemplifying the gating strategy is provided in the Supplementary Information.
